# Global Trends in Joint Arthroplasty: A Systematic Review and Future Projections

**DOI:** 10.3390/jcm14228214

**Published:** 2025-11-19

**Authors:** Bence Gusztáv Stubnya, Mercedes Schulz, Szilárd Váncsa, Gábor Sándor Szilágyi, Attila Szatmári, Zoltán Bejek

**Affiliations:** 1 Department of Orthopedics, Semmelweis University, Üllői Street 78/B, 2nd Floor, H-1082 Budapest, Hungary; 2 Centre for Translational Medicine, Semmelweis University, 22 Baross Street, H-1085 Budapest, Hungary; vancsa.szilard@semmelweis.hu; 3 Institute for Translational Medicine, Medical School, University of Pécs 12 Szigeti Street, H-7622 Pécs, Hungary; 4 Division of Pancreatic Diseases, Heart and Vascular Center, Semmelweis University, Tömő Street 25–29, H-1083 Budapest, Hungary

**Keywords:** arthroplasty, joint replacement, osteoarthritis, knee replacement, hip replacement

## Abstract

**Background/Objectives:** Osteoarthritis, the principal indication for joint arthroplasty, has doubled in incidence since 1990. Advances in surgical techniques, combined with an ageing population, have driven a substantial rise in arthroplasty procedures, presenting numerous challenges for healthcare systems worldwide. This study analyses historical trends in joint arthroplasty, assesses the accuracy of prior projections, and forecasts future demand using data from international arthroplasty registries. The goal is to guide healthcare resource management and policymaking. **Methods:** A systematic registry search identified 15 nations’ joint arthroplasty registries, and 209 annual reports provided high-quality data on primary total hip (THA) and knee arthroplasties (TKA) over the past two decades. Future arthroplasty volumes were projected using a deterministic negative-exponential saturating growth model. **Results:** From 2010 to 2023, THA incidence rose by 130% to 210%, while TKA increased by 150% to 664% across the analysed national registries. Projections to 2050 indicate sustained growth: THA volumes are expected to rise by 121% to over 200% and TKA by at least 130% across all countries. **Conclusions:** The ongoing escalation in joint arthroplasty demand necessitates proactive healthcare planning. Without strategic investments in infrastructure, workforce capacity, and digital resources, national healthcare systems risk being overwhelmed by the projected growth in both primary and revision procedures in the coming decades.

## 1. Introduction

The increasing need for and success of total joint arthroplasties (TJAs) have led to a significant rise, particularly in total hip (THA) and total knee arthroplasties (TKA) [1]. Beyond rising volumes, THA and TKA deliver large, sustained improvements in pain, function, and health-related quality of life and are generally cost-effective interventions, yet the increasing demand places a growing burden on healthcare systems. At the same time, the budget impact is substantial and varies by system; for example, a recent cross-country analysis reported a longer length of stay and relatively higher hospitalisation costs for primary THA in Austria than in Switzerland, even after adjustment for currency, purchasing power, and inflation [2,3,4].

Data from major national registries indicate a sharp increase in primary TJA procedures. In Australia, THA and TKA utilisation rose by 73% and 105%, respectively, from 2003 to 2013, with similar trends observed in the United States and Europe [5]. By 2030, TKA rates in the U.S. are projected to rise by 673%, while THAs will increase by 174% [6]. Contributing factors include an ageing population, rising obesity rates, and expanding surgical indications to younger patients. However, early interventions raise the likelihood of revision surgeries, as younger recipients outlive their implants. With more primary procedures being performed, revision arthroplasties are increasing, driven by periprosthetic joint infection (PJI), aseptic loosening, and mechanical failure. In Germany, revision TKA cases are expected to increase by 90% until 2050 [7]. Revision surgeries pose greater risks, longer hospital stays, and higher costs. In Australia, the financial burden of TKA is projected to reach AUD 1.38 billion by 2030, while the U.S. revision-related costs exceeded USD 1.62 billion in 2020 [5,8]. Beyond financial strain, the growing volume of surgeries is overwhelming healthcare providers. A shortage of orthopaedic surgeons and increasing workloads may lead to longer wait times, higher complication rates, and poorer outcomes. Additionally, postoperative care places a significant burden on caregivers, increasing stress and negatively impacting patient recovery.

## 2. Objectives

The primary aim of this study was to systematically analyse trends in joint arthroplasty procedures over the past few decades, with a particular focus on THA and TKA. By examining historical data, we assessed the increasing prevalence of these procedures, evaluated the burden of revision surgeries, and projected future trends based on existing evidence. To achieve this, we conducted an investigation of global trends in the number of primary and revision THA and TKA procedures over previous decades. A crucial aspect of this analysis involved the evaluation of revision surgeries.

## 3. Methods

**Figure 1:**
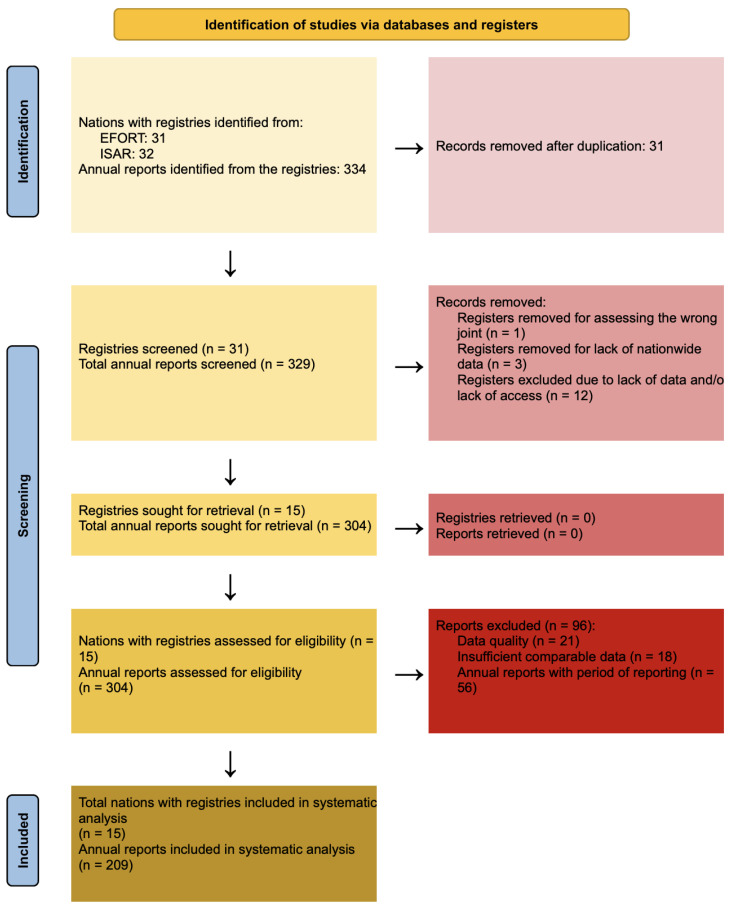
PRISMA flowchart.

We report our pre-registered systematic review on PROSPERO (Registration Number: CRD420250652207), based on the recommendation of the PRISMA 2020 guidelines (Figure 1), while following the Cochrane Handbook [9,10,11,12].

### 3.1. Eligibility Criteria

We only included national joint arthroplasty registries that reported comprehensive, systematically collected annual counts of total primary THA and TKA at the country level. Regional or institutional databases were excluded to ensure population-level representativeness. No language restrictions were applied provided that data extraction was feasible. Studies based on estimates, model-derived projections, or surveys—rather than observed registry counts—were excluded. Registries that reported only revision procedures without totals for all primary procedures or that exhibited incomplete or internally inconsistent reporting were also excluded. To minimise artefacts related to early capture and completeness run-in, model parameters were calibrated exclusively on the last contiguous pre-pandemic block ending in 2019 (preferentially 2010–2019; minimum ≥ 5 consecutive years). Early registry years are presented descriptively but were not used for model calibration. Registries lacking a ≥5-year contiguous block prior to 2020 were excluded from forecasting. Eligibility was operationalized across four prespecified domains, all of which had to be met for inclusion:

- National scope and coverage: nationwide capture (or a national mandate) with documented completeness sufficient for incidence/trend analysis (e.g., audit, statutory requirement, or published completeness estimates).
- Continuity of reporting: a clearly specified stretch of consecutive annual reports prior to 2020 adequate for modelling (recorded in years for each registry). No more than one missing year within any rolling five-year window was acceptable.
- Data granularity and consistency: availability of annual counts for primary and, where applicable, revision THA/TKA with stable procedure definitions over time or documented definitional changes amenable to harmonisation.
- Validation and quality controls: evidence of internal or external data quality procedures (e.g., cross-linkage, audit summaries, or formal data quality statements) described in registry documentation.

### 3.2. Information Sources

We systematically searched national joint arthroplasty registries and publicly available reports from government and healthcare organisations (updated search: 8 November 2025). To ensure comprehensive data collection, we consulted established national registries, if needed. Additionally, we examined reports from major orthopaedic associations, healthcare regulatory bodies, and published systematic reviews that referenced national registry data.

### 3.3. Search Strategy

A structured search strategy was employed on governmental and national orthopaedic societies’ databases to identify relevant registry reports and peer-reviewed publications containing national arthroplasty data.

### 3.4. Selection Process

All records retrieved from the searches were screened independently by two reviewers based on the predefined eligibility criteria. Discrepancies between reviewers were resolved through discussion or consultation with a third reviewer if necessary. The selection process was documented in a PRISMA flow diagram to illustrate the number of records identified, screened, assessed for eligibility, and included in the final analysis (Figure 1).

### 3.5. Data Collection Process

For each included registry, data was extracted using a standardised data collection form. Two independent reviewers systematically recorded key variables, and discrepancies were resolved through discussion. Data extraction focused on the total number of primary and revision THA and TKA procedures per year, demographic distributions, incidence of osteoarthritis, trends over time, and registry completeness. When necessary, corresponding registry representatives were contacted to clarify missing or unclear information.

### 3.6. Data Items

The primary data items collected included the annual number of THA and TKA procedures (both primary and revision), patient demographics (age and sex distribution), and registry coverage (percentage of national procedures captured). Where available, additional data on surgical indications, implant survival, revision rates, and revision causes were also extracted. To facilitate cross-country comparisons, data were standardised in terms of procedures per 100,000 population where possible.

### 3.7. Study Risk of Bias Assessment

The risk of bias assessment was performed by two independent authors using Cochrane’s Risk Of Bias In Non-randomised Studies of Interventions tool [13] (Supplementary Materials).

### 3.8. Synthesis Methods

We conducted a time series analysis to estimate and project the annual number of TKAs and THAs per 100,000 individuals from 2000 to 2050. Preceding data on TKA and THA incidence in national databases and previous publications, if incidence data were not available, we calculated the incidence rates using WHO population data [14,15]. The dataset included annual TKA and THA rates from 2000 to 2023, with missing values for certain years addressed via linear interpolation. We intentionally refrained from a pooled quantitative meta-analysis because substantial inter-registry heterogeneity could render summary estimates misleading.

To model temporal dynamics and project future TKA/THA rates, we used a deterministic negative-exponential saturating growth model (also termed an approach-to-asymptote model). The dependent variable was the annual rate per 100,000 population, with denominators derived from WHO annual population estimates. To avoid pandemic-related distortions, parameters were calibrated on the last contiguous pre-pandemic block ending in 2019 (preferentially 2010–2019), and the forecast origin was anchored at the observed rate for 2019 or 2023, depending on which was higher. The model’s initial growth was set by matching its slope to the pre-2020 compound annual growth rate estimated from the calibration window. We selected this specification because it preserves recent pre-pandemic growth in the short term while yielding plausible, non-explosive trajectories for longer time periods, and it is fully reproducible (closed-form parameterization; no stochastic components). Projections were generated for 2024–2050 using Python 3.13 (pandas/numpy/statsmodels/matplotlib). The forecast results were visualised graphically, with historical TKA/THA rates plotted alongside future projections.

## 4. Results

### 4.1. Total Knee Arthroplasty

A comprehensive analysis was conducted for fifteen countries—Australia, Canada, Czech Republic, Denmark, Finland, Germany, Hungary, New Zealand, Norway, Romania, Scotland, Sweden, Switzerland, the Netherlands, and the United Kingdom—for which robust, longitudinal data on annual TKA rates per 100,000 population or annual numbers were available.

Historical trends (2000–2023):

Across countries, incidence has generally increased over the past few decades, with a clear pandemic-associated decline around 2020, followed by partial to near-complete recovery by 2022–2023. Most jurisdictions showed higher latest-year rates than at baseline, although the magnitude of change varied (Table 1).

**Figure 2:**
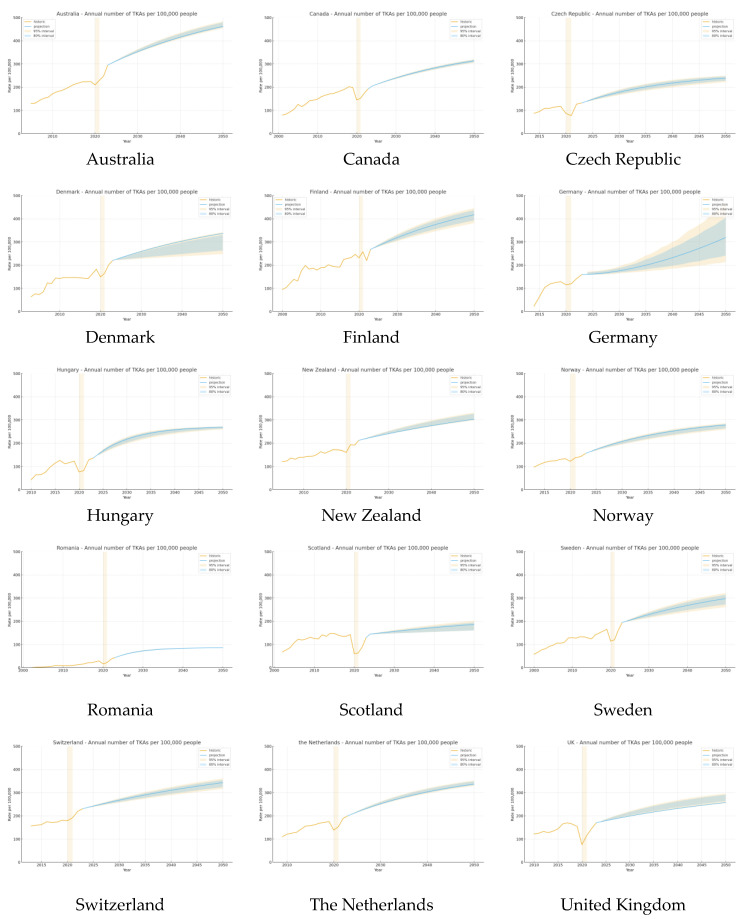
Historical and projected total knee arthroplasty (TKA) rates per 100,000 population by country. The solid yellow line shows the observed registry-based incidence, and the solid blue line represents the model-based projections. The light yellow-shaded area indicates the 80% uncertainty (prediction) interval, and the light blue-shaded area indicates the 95% uncertainty (prediction) interval around the projected rates. Yellow columns denote calendar years affected by the COVID-19 pandemic (2020–2021). Vertical axis scales are harmonised across country panels to facilitate visual comparability [17,18,19,20,21,22,23,24,25,26,27,28,29,30,31,32,33].

Model-based projections (2024–2050):

Our modelling, incorporating historical TKA rates, forecasts a continued rise in annual TKA utilisation through 2050 in all analysed countries. In Australia, the projected TKA rate is expected to surpass 400 per 100,000 by 2050. Similarly, Finland, Sweden, and Denmark are all projected to approach or exceed 300 per 100,000 by mid-century. Canada and New Zealand are estimated to reach values in the range of 250–300 per 100,000. The United Kingdom is projected to see a moderate but sustained increase, with rates forecast to reach 250 per 100,000 by 2050, while Romania is expected to continue its upward trajectory, potentially exceeding 80 per 100,000 by 2050, albeit from a lower baseline (Figure 2). 

For several countries, comprehensive statistical projections were not feasible due to insufficient temporal data or inconsistent registry reporting. However, available annual TKAs per 100,000 population rates provide a valuable perspective on longitudinal changes in arthroplasty utilisation.

Analysis of existing records reveals a consistent increase in TKA incidence across all observed countries. In Hungary, the annual number of TKAs per 100,000 population rose from 42.5 in 2010 to 126.2 in 2020, nearly tripling over the observation period. Similarly, Norway demonstrated a substantial increase, with TKA rates climbing from 97.0 per 100,000 in 2013 to 156.8 per 100,000 in 2023. Switzerland experienced a marked escalation as well, with the TKA rate rising from 156.6 in 2015 to 231.0 in 2023.

In Scotland, data available for two decades revealed a more than twofold increase in TKA rates, from 63.2 per 100,000 in 2002 to 147.6 per 100,000 in 2023. The Netherlands also exhibited a notable upward trajectory, with TKA rates increasing from 110.6 in 2015 to 168.8 in 2023. The Czech Republic saw TKA rates rise from 77.1 per 100,000 in 2015 to 126.9 per 100,000 in 2022.

Taken together, these trends indicate that the annual number of TKAs has increased substantially in all countries for which sufficient longitudinal data were available, consistent with regional and global trends in the burden of osteoarthritis, as the main indication for the adoption of arthroplasty procedures.

### 4.2. Total Hip Arthroplasty

**Figure 3:**
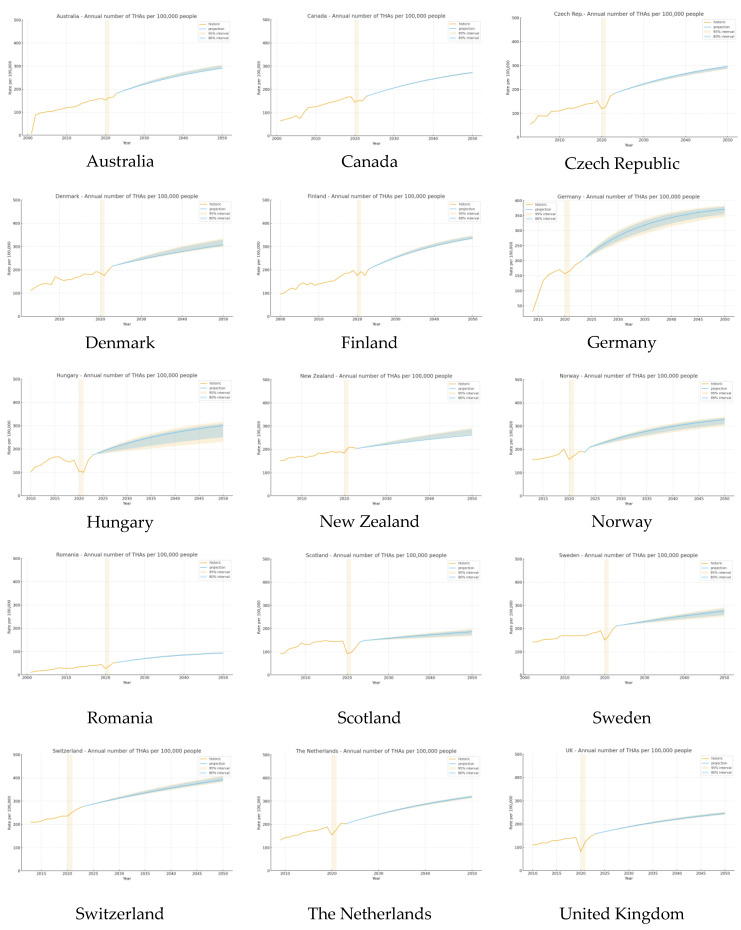
Historical and projected total hip arthroplasty (THA) rates per 100,000 population by country. The solid yellow line shows the observed registry-based incidence, and the solid blue line represents the model-based projections. The light yellow-shaded area indicates the 80% uncertainty (prediction) interval, and the light blue-shaded area indicates the 95% uncertainty (prediction) interval around the projected rates. Yellow columns denote calendar years affected by the COVID-19 pandemic (2020–2021). Vertical axis scales are harmonised across country panels to facilitate visual comparability [17,19,20,21,22,23,24,25,26,27,28,29,30,31,32,33,34].

Historical trends (2000–2022/2023):

Analysis of national registry data reveals a marked increase in THA procedures in all countries over the past two decades, although the COVID-19 pandemic led to a temporary decline in procedure volumes in 2020 in almost every registry examined. In most countries, annual THA counts rebounded after the pandemic; in many cases, the most recent data (2023) have returned to the pre-pandemic peaks observed in 2019 (Figure 3).

Model-based projections (2024–2050):

Our modelling for countries with robust longitudinal data and scenario-based estimation for Germany projected that the annual number of THA procedures will continue to increase until 2050. In Australia, the number of THA procedures is projected to reach approximately 300 per 100,000 people by 2050, representing a further substantial increase over current volumes. Similar upward trajectories are forecasted for Finland, Sweden, Denmark, and Canada, with projected THA procedure counts in 2050 anticipated to exceed more than 1,5 times their latest pre-pandemic levels. New Zealand and the United Kingdom are expected to experience more moderate, yet still significant, increases. For Germany, based on clinical consensus and demographic factors, a scenario was modelled in which the annual number of THA procedures increases by 30–70% by 2050, compared to 2023 volumes (Figure 3).

Overall, projections indicate that the substantial growth in THA utilisation observed in the past decades will continue for the foreseeable future. The rate and magnitude of future increases are expected to vary according to baseline utilisation, population ageing, and national health system capacity, but the general trend toward rising demand for hip arthroplasty is consistent across all high-income countries with available data.

## 5. Discussion

Osteoarthritis (OA), colloquially referred to as the “wear and tear” disease, predominantly affects the elderly and is increasing in prevalence globally. As of 2019, OA had a global prevalence of 7.09% and an incidence rate of 536 per 100,000 individuals [14]. This condition significantly impacts quality of life, leading to chronic pain and disability; thus, it is a major contributor to the loss of healthy life years [15,35]. Consequently, joint arthroplasties, particularly THAs and TKAs, have become the most successful treatment options [1]. These procedures have not only increased in frequency but are projected to rise further due to the ageing population and durability challenges of prosthetic components, but also due to the increasing global prevalence of osteoarthritis.

### 5.1. Total Knee Arthroplasty

Concurrently, younger patients are increasingly undergoing arthroplasty procedures. As observed in our study, annual TKA procedures are rising globally. For instance, the prevalence of TKA in Finland rose from 1.1% in 2000 to 2.6% in 2010 and 4.0% in 2020, reflecting a 270% rise between 2000 and 2020 and a 56% increase from 2010 to 2020. The total number of TKA implants escalated from 7535 in 1990 to 34,884 in 2000 and further to 162,711 in 2020. In Hungary, the annual number of primary knee arthroplasties tripled between 2010 and 2023, from 4241 to 13,240.

The future burden of TJA is immense, with a notable increase in revision surgeries due to the ageing demographic. Over the last two decades, several studies have predicted a surge in primary procedures; some of the different projections estimate a surge of up to 250% in the need for total knee prosthetic implants [7,36,37,38,39,40]. These projections can now be re-evaluated by accounting for the reduction in surgical volumes during the pandemic, thereby enabling a comparison between predicted and observed trends.

One of the most impactful studies from this century by Kurtz et al. projected a 673% increase in primary TKA demand in the United States [6]. According to Inacio et al., an increase of 143% from 2012 to 2050 is expected, reaching approximately 1.5 million cases per year by mid-century [39]. Further projections, based on the 2014 National Inpatient Sample (NIS) data, estimate that the total annual utilisation of primary TKA in the United States will increase by 110% in 2025, 182% in 2030, and 401% by 2040 [38]. In 2008, 615,050 TKA procedures were performed in the United States, representing a 134% increase from 1999, while the population grew by only 11%. Notably, the number of TKAs more than tripled among individuals aged 45 to 64, despite a 29% population growth in this age group [41]. Regardless of the projection model used, a clear rise in knee arthroplasties is expected in the United States, with the lifetime risk of primary TKA estimated at 7% for males and 9.5% for females [42].

In Germany, between 2008 and 2018, there was a 46.3% increase in primary knee arthroplasties among patients younger than 65 years [43]. Between 2005 and 2018, Germany recorded a total of 2,151,448 primary TKAs. During this period, the annual number of TKA procedures increased from 128,932 to 170,494, representing a 32.4% increase. These trends place Germany among the leading countries in the prevalence of TKA, with a projected increase of 43% by 2050, reaching an incidence rate of 299 per 100,000 people [7].

Australia has observed a 105% increase in the utilisation of TKA procedures. In 2003, the TKA rate was 123 per 100,000 population, rising to 213 per 100,000 population by 2013 [5]. This trend is projected to increase to more than 350 per 100,000 as predicted by our study model. The most significant absolute growth occurred among individuals aged 40–69 years, while the number of procedures for those under 40 years remained relatively unchanged. The total cost of TKA escalated from AUD 448 million in 2003 to AUD 905 million in 2013, with the incidence of TKA (for OA) projected to rise by 276% by 2030—potentially creating an unsustainable burden on the healthcare budget and workforce [5].

Hamilton et al. estimated that the lifetime risk of knee arthroplasty in the UK is approximately 10%, with the rates continually rising due to increased longevity and eventual prosthesis failure [44]. The most significant growth in TKA cases is expected among those aged 40–69, with a 269% increase projected for 40–49 year-olds, a 94% increase for 50–59 year-olds, and a 43% increase for 60–69 year-olds [44]. These trends highlight the multifaceted reasons behind earlier joint failures, including lifestyle factors and the physical demands on younger patients.

### 5.2. Total Hip Arthroplasty

Analysing our historical data, the growth rates in THA procedures were highest in Central and Western European countries such as Switzerland, Germany, and Hungary. Scandinavian countries also showed a continued increase in THA incidence, although the pace was slower than expected when compared with other economically similar nations. By 2050, the highest projected incidence rates for THA—approaching 300 per 100,000—are expected in high-income countries, particularly in Oceania, Finland, Germany, and Canada.

As anticipated, procedural volumes visibly declined during the COVID-19 pandemic years of 2020 and 2021, with operative volume recovering over the following two years. Notably, Switzerland was the only country in our study that maintained high procedural volumes during the pandemic, even showing a slight increase in annual THAs during this period (19,897 in 2019, 20,215 in 2020, and 21,815 in 2021).

In 2007, Kurtz et al. predicted an increase in primary THA volumes of 174%, translating to a procedural volume of 572,000 by 2030 in the United States [6]. Between 2014 and 2023, an increase of 114% was observed, suggesting that the predicted percentage growth in procedural volume may remain realistic despite pandemic-related disruptions. However, the American Joint Replacement Registry (AJRR) reports 140,974 annual primary THA procedures for 2023, raising doubts about whether volumes will reach 572,000 by 2030 [42]. Schichman et al. project a growth to at least 624,766 cases by 2040, whereas Dubin et al. assume a slowing projection, possibly due to financial limitations, thus estimating a growth of only 10% by 2060 [45,46].

Recent forecasts for Australia suggest a probable annual procedural incidence rate of 302 per 100,000 by 2030 when assuming a progressive rise in yearly operative volumes. This reflects a predicted increase of 208% compared to 2013 [5]. A further study estimates an incidence rate of 510 per 100,000 for ages 40+ by 2046, which is comparable to our predictive value of 260 per 100,000 regarding the whole Australian population [47]. Similarly to Dubin et al., Inacio et al. predict a reduction in growth from 2% annually by 2025 to 1% annually by 2046, suggesting that growth rates may eventually plateau [39,47].

Romania have experienced a 4-fold increase in THA volume over the past two decades, with an observed rise of 8.21% in THAs between 2001 and 2019. When adjusting this rate to a substantial population decline of almost 3 million, procedural rates may be considerably higher. The most recent projection suggests that these rates could possibly double by 2034, in line with our calculated projection of a doubling rate between 2024 and 2050 [48].

Germany has demonstrated one of the fastest growth rates for primary THAs, with an increase of 143% from 2015 to 2023. The most recent ERPD publication reported 166,624 procedures performed in 2023, with incidence rates expected to reach 360 per 100,000 by 2040—totalling approximately 288,000 procedures [49]. A study by Rupp et al. projected 282,034 annual THAs by 2040 (95% CI 230,473 to 345,228), aligning with this estimate [50]. The average annual increase between 2016 and 2023 was roughly 6%. If this trend continues, over 450,000 procedures could be performed by 2040, significantly exceeding earlier predictions from 2018 [49].

We deliberately restricted the analyses to primary THA/TKA. Cross-national modelling of revision arthroplasty lies outside the scope of this work because revision metrics are highly contingent on health-system and registry-process characteristics (access to care, capture of outpatient procedures, registry coverage and longitudinal follow-up, linkage completeness) and are further complicated by heterogeneity in definitional frameworks (e.g., revision vs. re-operation; indication coding). In the absence of rigorous harmonisation, these differences risk conflating clinical signals with ascertainment and reporting artefacts, thereby compromising cross-country comparability and interpretability.

To assess the future burden of the increasing procedural volumes, it is essential to evaluate the capacity of healthcare systems to manage this workload—particularly the availability of trained surgeons, which remains a key limiting factor. The American Academy of Orthopaedic Surgeons (AAOS) reported an average of 65.2 arthroplasties per orthopaedic surgeon in 2017 [51]. If current caseload trends continue, this number is projected to rise to 139.4 procedures per surgeon in 2050 [51,52]. However, current data indicates that the average age of surgeons in the United States is reaching 62.4 years, suggesting that retirements may significantly impact the availability of practitioners [42]. Although surgeon training has expanded globally in response to rising population demands, the growth is disproportional to that of the increasing procedural volumes. During our study period, the number of orthopaedic surgeons increased by a mean of 51% (52 for THA, 50.3 TKA) in New Zealand, 46.8% (23.6 THA, 70 TKA) in the United States, and 43.9% (51 THA, 36.7 TKA) in the United Kingdom, none of which match the percentage increase in surgeries [7,24,33,53]. Without drastic efforts to increase the number of surgeons in training, governmental healthcare systems risk facing major capacity shortages and exhausted systems in the future.

Shifts in public health, particularly rising obesity rates, which contribute to the earlier onset of osteoarthritis, are driving the increased demand for arthroplasty procedures, particularly among younger patients. Younger patients are undergoing as many arthroplasties as ever—over 1.45 million adults aged 50–69 in the U.S. have a primary TKA, with an additional 178,000 having already had a revision [7,18,33,42]. Given the average lifespan, those receiving arthroplasties in their 50s are more likely to require revisions, especially as higher activity levels accelerate prosthetic wear and increase the risk of complications. Evans et al. analysed TKA survival rates and found that approximately 18% of procedures required revision within 25 years, thus significantly contributing to the increasing procedural volume [1]. Once revised, the lifespan of the implant shortens significantly. In the United Kingdom, 19.6% of hips revised within one year are re-revised within ten, adding even more procedures to an already overloaded healthcare system [54].

A continued rise in demand for joint arthroplasty procedures, alongside ageing populations and unprepared healthcare infrastructure, is expected to lead to longer waiting lists and a decline in patients’ quality of life (QoL). Clement et al. revealed that an additional waiting period of 6 months was associated with significant clinical deterioration in health-related QoL [55]. Underlying conditions such as osteoarthritis and obesity may progress, increasing the risk of postoperative complications and potentially necessitating earlier revision procedures [55]. The rise in revision surgeries places additional strain on healthcare systems, further supporting the need for expanded surgical training programmes to meet future demand. COVID-19 has contributed to these challenges, as previously planned surgeries require rescheduling—a process that, at optimal rates, is estimated to take 24–27 months [55]. Moreover, patients with more favourable outcomes are often prioritised, leading to prolonged delays and deterioration of high-risk patients, who may ultimately become ineligible for surgery.

In light of this, robotic-assisted surgery may prove beneficial in increasing operative volume, reducing surgeon strain, and improving overall workforce load. With its introduction into the clinical world, it has been proven to improve the accuracy of implant placement and shorten postoperative recovery times, improving long-term outcomes overall and ultimately prolonging implant survival. The successful integration of robotic devices requires considerable investment in technology and surgeon training. In the long term, robotic surgery may have the capability to mitigate the potential burden of increased surgical demand by reducing physical strain on surgeons, as well as reducing the need for follow-up surgeries, thus counteracting the increasing incidence rates of revision arthroplasties. Further research is needed to evaluate these long-term effects and to determine whether robotic surgery can effectively reduce operating time, improve clinical outcomes, and ease the workload on surgeons [56,57,58].

## 6. Strengths and Limitations

A key strength of this study is the use of national registry data spanning a large study period (2000–2023), which provides reliable continuous data for the assessment of procedure rates over time and the estimation of their projected growth.

Several limitations were identified. Most notably, the use of databases from multiple countries introduces variability in data collection methods and reporting accuracy. There may be cross-national variability in the definitions of reported procedures, especially in distinguishing between reoperations and revisions of arthroplasty, which may affect data comparability. Differences in reporting systems—particularly the use of paper-based methods versus online reporting software—may influence annual completeness rates. Digital systems are generally superior, as they prevent data loss and establish standardised reporting practices. While most registries have adopted electronic data submissions, resulting in completeness rates above 90%, earlier records often show major data gaps, possibly limiting the comparability of procedure rates over the past two decades.

In sensitivity checks comparing registry-based incidence with OECD Health at a Glance 2023 [16], estimates were frequently non-concordant. Across 75 country–year pairs spanning 14 countries, 54/75 (72%) comparisons differed by >10%, and 13/14 countries had at least one >10% discrepancy (Canada was the sole exception; no OECD comparator was available for Scotland). The mean absolute gap was ~21% for TKA and ~37% for THA. The largest OECD-above-registry differences occurred for Germany (e.g., THA-2019 315 vs. 170 per 100,000; +85%) and Romania (e.g., THA-2011 52 vs. 28; +86%), whereas registry rates exceeded OECD in New Zealand (e.g., TKA-2021 193 vs. 103; −47%) and Hungary (TKA-2021 82 vs. 28; −66%). These divergences likely reflect differences in numerator construction (e.g., inclusion of revisions, counting of bilateral procedures, capture of private/ambulatory activity, registry coverage and follow-up) and denominator definitions (resident vs. treated population) (Table 2).

During the study period, several registries updated their reporting methods, often altering the type of procedures reported (e.g., total hip arthroplasty vs. hip arthroplasties), the reporting period (calendar year vs. fiscal year), and/or the classification standards used to define the inclusion criteria. In some registries, earlier datasets were reviewed and revised after being judged inappropriate due to the limitations of paper-based reporting and their consequent errors [42]. Retrospective modifications resulted in new classification approaches, ultimately resulting in the exclusion of substantial numbers of procedures from annual reports. Additionally, registries that operate as continuously updated online databases allow for the correction of data gaps over time, in contrast to those relying on fixed annual publications. This difference in reporting methods may lead to inconsistencies in annual procedure rates between the publication formats. As a result, the data presented in this study may not accurately reflect annual changes in a manner that is directly comparable across the registries. We did not perform meta-analytic pooling because of substantial inter-registry heterogeneity (case definitions, reporting frames, completeness/coverage), which precludes a methodologically defensible quantitative synthesis.

Despite our efforts to compare global trends, most public arthroplasty registers originate from Europe, with additional databases from North America and Oceania. Disappointingly, South America and Africa have very limited arthroplasty registry reportage, while records in Asia are largely restricted to members affiliated with the registry association.

## 7. Conclusions

The global increase in primary and revision THA and TKA procedures imposes a substantial and growing burden on healthcare systems, particularly in the context of limited workforce expansion. Although some earlier projections may have overestimated absolute procedure numbers, incidence rates continue to rise, indicating a sustained and clinically relevant demand. Our findings demonstrate consistent annual growth in procedure volumes across multiple countries, in line with global trends in osteoarthrosis burden and increasing life expectancy. The future workload will be increasingly driven by revision surgeries, further amplified by population ageing, rising obesity rates, and the implantation of prostheses at younger ages.

While this study provides a comprehensive overview of international arthroplasty trends, its interpretation is constrained by heterogeneity and inconsistencies in registry data across countries. To address this, we strongly advocate for the development of an international consensus on data collection and reporting standards in national arthroplasty registries to enhance transparency, comparability, and reproducibility. Robust, harmonised data are essential for defining clinical priorities and supporting evidence-informed healthcare planning. In parallel, as the incidence of both primary and revision procedures continues to increase, targeted investments will be required to expand surgical capacity and allocate resources efficiently. Further comparative research is warranted to determine which health systems and patient groups will require the most urgent and substantial reinforcement.

Taken together, the observed and projected trends in THA and TKA should be viewed as a clear signal for anticipatory health policy and workforce planning. Proactive investment in operative infrastructure, perioperative and rehabilitation services, and the expansion of arthroplasty-focused surgical training will be crucial to prevent widening gaps between procedural demand and service provision. At the same time, international harmonisation and strengthening of arthroplasty registries are needed to ensure timely, comparable data for monitoring access, outcomes, and equity. Coordinated policy, workforce, and data strategies will be essential to sustain high-quality arthroplasty care in the face of rapidly rising procedural volumes.

## Figures and Tables

**Table 1:** Difference in arthroplasty incidence (% change) over selected intervals based on registry data. Percent change in incidence per 100,000 for THA and TKA across three intervals: 2015→2019, 2015→2023, and 2019→2023 [16].

	Australia	UK	Canada	Germany	Sweden	Hungary	Finland	Denmark	Romania	New Zealand	Switzerland	Scotland	The Netherlands	Czech Republic	Norway
**THA**															
2015–19	111%	111%	114%	incomplete data	114%	92%	119%	113%	124%	104%	110%	99%	112%	116%	124%
2015–23	128%	123%	115%	incomplete data	125%	106%	124%	127%	149%	112%	129%	95%	120%	139%	116%
2019–23	115%	111%	101%	116%	110%	115%	104%	112%	121%	108%	118%	96%	107%	120%	93%
TKA															
2015–19	106%	107%	111%	incomplete data	134%	108%	129%	126%	189%	107%	112%	97%	111%	124%	114%
2015–23	140%	118%	108%	incomplete data	157%	120%	140%	152%	284%	135%	142%	88%	127%	140%	134%
2019–23	132%	110%	97%	124%	117%	111%	109%	120%	150%	126%	128%	91%	114%	113%	117%

**Table 2:** Comparison of the OECD and registry data. Bold green marks the higher of the paired values (OECD vs. registry), and red-shaded cells denote an absolute OECD–registry discrepancy of ≥10%.

Country	TKA—2011 OECD/Registry	TKA—2019 OECD/Registry	TKA—2021 OECD/Registry	THA—2011 OECD/Registry	THA—2019 OECD/Registry	THA—2021 OECD/Registry
Australia	178/**180**	203/**224**	**252**/229	**171**/122	**171**/160	**198**/164
Canada	**161**/159	198/**199**	152/152	**131**/129	**168/168**	**153**/152
Czech Republic	110/n.a.	**121**/117	**108**/77	**160**/116	**173**/152	**198**/126
Denmark	**169**/146	**203**/182	**173**/164	**227**/154	**259**/192	**236**/174
Finland	**193**/189	242/**246**	**260**/258	**225**/143	**280**/196	**284**/192
Germany	210/n.a.	**227**/128	**201**/119	290/n.a.	**315**/170	**301**/166
Hungary	55/**64**	94/**123**	28/**82**	113/**124**	147/**162**	79/**100**
New Zealand	93/**143**	109/**168**	103/**193**	142/**165**	160/**189**	147/**208**
Norway	89/n.a.	117/**134**	122/**138**	243/n.a.	**268**/201	**255**/173
Romania	**13**/7	27/**28**	18/**21**	**52**/28	**77**/44	**65**/38
Scotland	n.a./123	n.a./142	n.a./63	n.a./131	n.a./146	n.a./96
Sweden	**129**/127	141/**165**	100/**120**	**238**/169	**249**/191	**215**/167
Switzerland	214/n.a.	**260**/181	**273**/191	300/n.a.	**313**/235	**323**/252
The Netherlands	**137**/125	155/**175**	n.a./153	**226**/145	**254**/188	n.a./178
UK	**140**/125	142/**154**	108/**115**	**177**/113	**182**/142	**180**/125

## Data Availability

The datasets used in this study can be found in the full-text articles included in the systematic review and meta-analysis.

## Supplementary Materials

The following supporting information can be downloaded at: https://www.mdpi.com/article/10.3390/jcm14228214/s1, PRISMA 2020 Checklist; Table S1: Risk of bias assessment; Table S2: Study characteristics.

## Abbreviations

AUD	Australian dollars (AUD) at first use.
CI	Confidence interval (CI).
COVID-19	Coronavirus disease 2019 (COVID-19).
EPRD	German Arthroplasty Registry (Endoprothesenregister Deutschland, EPRD) at first mention.
OECD	Organisation for Economic Co-operation and Development (OECD).
PRISMA	Preferred Reporting Items for Systematic Reviews and Meta-Analyses (PRISMA).
PROSPERO	International Prospective Register of Systematic Reviews (PROSPERO).
THA	Total hip arthroplasty (THA).
TJA	Total joint arthroplasty (TJA).
TKA	Total knee arthroplasty (THA).
UK	United Kingdom (UK).
USD	US dollars (USD).
WHO	World Health Organization (WHO).
